# Aggrecan: a new biomarker for acute type A aortic dissection

**DOI:** 10.1038/s41598-021-89653-y

**Published:** 2021-05-14

**Authors:** Karl C. König, Harald Lahm, Martina Dreßen, Stefanie A. Doppler, Stefan Eichhorn, Nicole Beck, Kathrin Kraehschuetz, Sophia Doll, Stefan Holdenrieder, Adnan Kastrati, Rüdiger Lange, Markus Krane

**Affiliations:** 1 Division of Experimental Surgery, Department of Cardiovascular Surgery, Institute Insure, German Heart Center Munich, Lazarettstrasse 36, 80636 Munich, Germany; 2 Department of Proteomics and Signal Transduction, Max Planck Institute of Biochemistry, Martinsried, Germany; 3 Institute of Laboratory Medicine, German Heart Center Munich, Munich, Germany; 4 DZHK (German Center for Cardiovascular Research) – Partner Site Munich Heart Alliance, Munich, Germany; 5 Department of Cardiology, German Heart Center Munich, Munich, Germany

**Keywords:** Biomarkers, Diagnostic markers, Aortic diseases, Diagnostic markers

## Abstract

Acute type A aortic dissection (ATAAD) constitutes a life-threatening aortic pathology with significant morbidity and mortality. Without surgical intervention the usual mortality rate averages between 1 and 2% per hour. Thus, an early diagnosis of ATAAD is of pivotal importance to direct the affected patients to the appropriate treatment. Preceding tests to find an appropriate biomarker showed among others an increased aggrecan (ACAN) mRNA expression in aortic tissue of ATAAD patients. As a consequence, we investigated whether ACAN is a potential biomarker for diagnosing ATAAD. Mean ACAN protein concentration showed a significantly higher plasma concentration in ATAAD patients (38.59 ng/mL, n = 33) compared to plasma of patients with thoracic aortic aneurysms (4.45 ng/mL, n = 13), patients with myocardial infarction (11.77 ng/mL, n = 18) and healthy volunteers (8.05 ng/mL, n = 12). Cardiac enzymes like creatine kinase MB and cardiac troponin T showed no correlation with ACAN levels in ATAAD patients. Receiver-operator characteristics (ROC) curve analysis for ATAAD patients versus control subjects an optimum discrimination limit of ACAN plasma levels at 14.3 ng/mL with a corresponding sensitivity of 97% and specificity of 81%. According to our findings ACAN is a reliable potential biomarker in plasma samples to detect ATAAD with high sensitivity and specificity.

## Introduction

Acute type A aortic dissection (ATAAD) is a life-threatening diagnosis which is associated with significant morbidity and mortality^1^. After onset of symptoms patients suffering from ATAAD have an associated mortality rate of 1–2% per hour during the first 48 h without surgical intervention. This value increases to 80% after 14 days and to 90% after three months^2^. However, a correct early diagnosis is complicated by the rare frequency of ATAAD. The incidence of ATAAD varies between five and 30 cases per million and year in the United States depending on the prevalence of risk factors^2–4^. Furthermore, ATAAD shares similar symptoms with more common clinical presentations such as myocardial infarction, vascular embolization, gastric ulcer or acute back pain^5^. Atypical presentation generally bears the risk of missing the truly underlying disease process. The risk of missing an ATAAD is about 40%^2^. Definitive management is often delayed for several hours until the diagnostic process is completed. Seventy five percent of ATAAD patients are admitted into non-specialized hospitals^2,6^. The time to hospital is often at least one to two hours after the onset of symptoms. The mean time to treatment or at least definitive diagnosis is more than six hours in Europe and exceeds 15 h in the USA according to the IRAD investigators^2,4,6^. Hence, a clear and early diagnosis of ATAAD is crucial for immediate surgical intervention resulting in an improved survival rate for the patient. Therefore, the establishment of a specific and sensitive blood biomarker for diagnosing ATAAD would be the key to reduce the time period between symptom onset and the essential surgical treatment.

Within the last decades several biomarkers have been established in cardiovascular medicine. Especially creatin kinase MB (CK-MB) and cardiac troponins (cTnTs) have a long history for the specific and sensitive detection of myocardial infarction (MI)^7,8^. In addition, we have recently described MYBPHL as a reliable biomarker to specifically predict damage of atrial tissue^9^. Although preliminary data suggest a possible role of plasma biomarkers, like smooth muscle derived calponins^10^, myosin heavy chain^11^ or the fibrin fragment D-dimer^12^ in early diagnosis of ATAAD, at present no reliable biomarker exists, to diagnose ATAAD with sufficient specificity and sensitivity.

Aggrecan (ACAN) is a multimodular proteoglycan which can make up to 10% of the cartilage^13^ ACAN plays a major role in bone and cartilage morphogenesis and several mutations have been identified in patients with short stature^14^. However, analysis of the proteoglycanome confirmed the presence of ACAN in the normal human aorta and also in aortic lesions of ATAAD patients^15^.

Here we show that ACAN protein levels are significantly enhanced in plasma samples of ATAAD patients compared to samples from healthy controls. In addition, plasma ACAN levels of several clinical control cohorts stayed far beyond the values obtained in ATAAD patients and were similar to the concentration of healthy controls. Thus, our data suggest that ACAN may be a useful new biomarker for early diagnosis of ATAAD.

## Methods

### Blood samples and biopsies

Between February 2017 and January 2020 105 Patients underwent emergency surgical repair for ATAAD at our institution. Blood samples of 33 non-consecutive hemodynamically stable ATAAD patients, who consented to take part in our study, were collected during that period. Hemodynamically unstable patients were excluded due to the impossibility to obtain an informed consent. Seventy-two patients were not included during the given time period. Of these patients, three suffered from a major stroke preoperatively as part of the ATAAD. Five of these patients were hemodynamically unstable, one of them already connected to the ventilator at the time of admission. 64 patients were not successfully consented preoperatively. Samples were drawn directly after admission and centrifuged at 2000×*g* for 10 min at 4 °C. Plasma was partitioned in 200 µL aliquots and immediately stored at − 80 °C within 30 min after admission until further use. Basic demographic and specific pathological data for all ATAAD patients are shown in Table 1. Plasma samples from all other experimental cohorts (Supplementary Table S1) were supplied by the cardiovascular biobank at the German Heart Center Munich (KaBi-DHM). Human biopsies (skeletal muscle, fat tissue, left atrium, aortic tissue from ATAAD or coronary artery bypass graft patients, *Vena saphena magna* and *Arteria mammaria interna*) (Supplementary Table S2) were obtained during surgical procedures, directly snap-frozen and stored in liquid nitrogen until further use.

**Table 1:** Baseline characteristics and specific pathological features of patients with ATAAD.

Characteristics (n = 33)	n (%)
Age, mean (SD), years	65 ± 12.1
**Sex**	
Male	15 (45.5)
Female	18 (54.5)
**Left ventricular ejection fraction**	
Normal	31 (94)
Slightly reduced	2 (6)
Moderately reduced	0
Severely reduced	0
Coronary artery disease	6 (18)
Peripheral arterial disease	1 (3)
Carotid artery disease	1 (3)
**Cardiovascular risk factors**	
Arterial hypertension	27 (82)
Hyperlipidemia	10 (30)
Diabetes mellitus	5 (15)
Obesity	5 (15)
Nicotine abuse	12 (36)
Family disposition	5 (15)
COPD	1 (3)
Chronic kidney disease	1 (3)
Prior stroke	3 (9)
Prior myocardial infarction	1 (3)
Osteoarthritis	0
Prior cardiac surgery	1 (3)
**Stanford classification**	
Type A	33 (100)
Type B	0
**DeBakey classification**	
Type I	20 (60.6)
Type II	13 (39.4)
Type III	0
**Preoperative false lumen status**	
Patent	33 (100)
Partially thrombosed	0
Totally thrombosed	0
Preoperative lower extremity ischemia	1 (3)
Prior thoracic aortic aneurysm	20 (60.4)
Mean aortic aneurysm size (SD)	40.8 mm ± 23.5
Aortic valve disease	16 (48.5)
Aortic stenosis	0
Aortic regurgitation	16 (48.5)
AR I°	7
AR II°	6
AR III°	3
Bicuspid aortic valve	1 (3)
**Underlying syndrome**	
Marfan	2 (6)
Ehlers Danlos	0
Loeys-Dietz	0
Turner	0
Iatrogenic aortic dissection	1 (3)

*ATAAD* acute type A aortic dissection, *SD* standard deviation, *n* number of patients, *%* percent of patients, *COPD* chronic obstructive lung disease, *y* years, *AR* aortic regurgitation.

All procedures and sampling were approved by the local ethics committee of the Medical Faculty at the Technical University of Munich (Project nos. 5943/13 and 223/18S). All samples in the KaBi-DHM were obtained with informed consent signed by all participants or probands or their legal guardians prior to the inclusion in the study. All study procedures were performed in accordance with relevant guidelines and regulations and they conformed to the ethical standards of the Declaration of Helsinki.

### Protein expression in different heart regions

Protein concentrations in different heart regions had been previously determined by mass spectrometry^16^.

### Assessment of gene expression in human biopsies by qRT-PCR

Frozen biopsies were homogenized in 900 µL QIAzol lysis reagent for 30 s using an Ultraturrax MICCRA D-8 (ART Moderne Labortechnik, Müllheim, Germany) and processed with the RNeasy Plus Universal Mini Kit (QIAGEN, Hilden, Germany) according to the manufacturer’s recommendation. One hundred ng total RNA were reverse-transcribed into cDNA with M-MLV reverse transcriptase (150 U, Invitrogen, Carlsbad, CA), random hexamer primers (375 ng), dNTPs (10 mM each), 10 mM DTT and 1 × first strand buffer in a final volume of 30 µL for 50 min at 37 °C. The enzyme was inactivated for 15 min at 70 °C. Gene-specific amplification of 1 µL cDNA was performed on a Quant Studio 3 (ThermoFisher, Dreieich, Germany) with 0.3 µM of each primer and Power SYBR Green Mastermix (ThermoFisher) using the following cycling conditions: 95° for 10 min to activate *Taq* polymerase, followed by 40 cycles of 95 °C for 15 s and 60 °C for 60 s. The relative gene expression was normalized to *ACTB* (*β-Actin*) expression as the reference. The sequences of all primers are shown in Supplementary Table S3.

### Measurement of ACAN, OGN and ITGA11 in plasma samples by ELISA

Commercially available ELISA kits were used to determine the concentration of aggrecan (ACAN) (Cat.No. SEB908Hu, Cloud Clone Corp., Katy, TX), osteoglycin (OGN) (Cat.No. LS-F22608, LifeSpan Biosciences Inc., Seattle, WA) and integrin α 11 (ITGA11) (Cat.No. CSB-EL011863HU, Cusabio, Houston, TX) in plasma samples according to the manufacturers’ instructions. In brief, all components and samples were brought to room temperature and 100 µL of undiluted plasma samples were added, processed and plates were read at 450 nm. In each assay a standard curve was included to determine the concentration in individual samples.

### Statistical analysis

Data distribution was assessed using the Shapiro–Wilk test. Differences in gene expression were determined by the Mann–Whitney Rank Sum test or the one-way-ANOVA test. Significance of differences in ACAN protein concentrations for multiple groups was estimated by the Wilcoxon-Mann–Whitney, Kruskal Wallis or one-way-ANOVA test by all pairwise multiple comparison procedures (Dunn’s or Holm-Sidak Method). The sensitivity and specificity were analyzed applying the receiver-operator characteristics (ROC) curve analysis. Positive and negative predictive values were calculated using the Chi-square test. Significance is indicated as *p < 0.05, **p < 0.01, ***p < 0.001. Values are presented as mean ± standard error of the mean (SEM), 95% confidence interval (CI) and fold change as appropriate.

## Results

### Selection of candidate genes to diagnose acute type A aortic dissection

**Figure 1:**
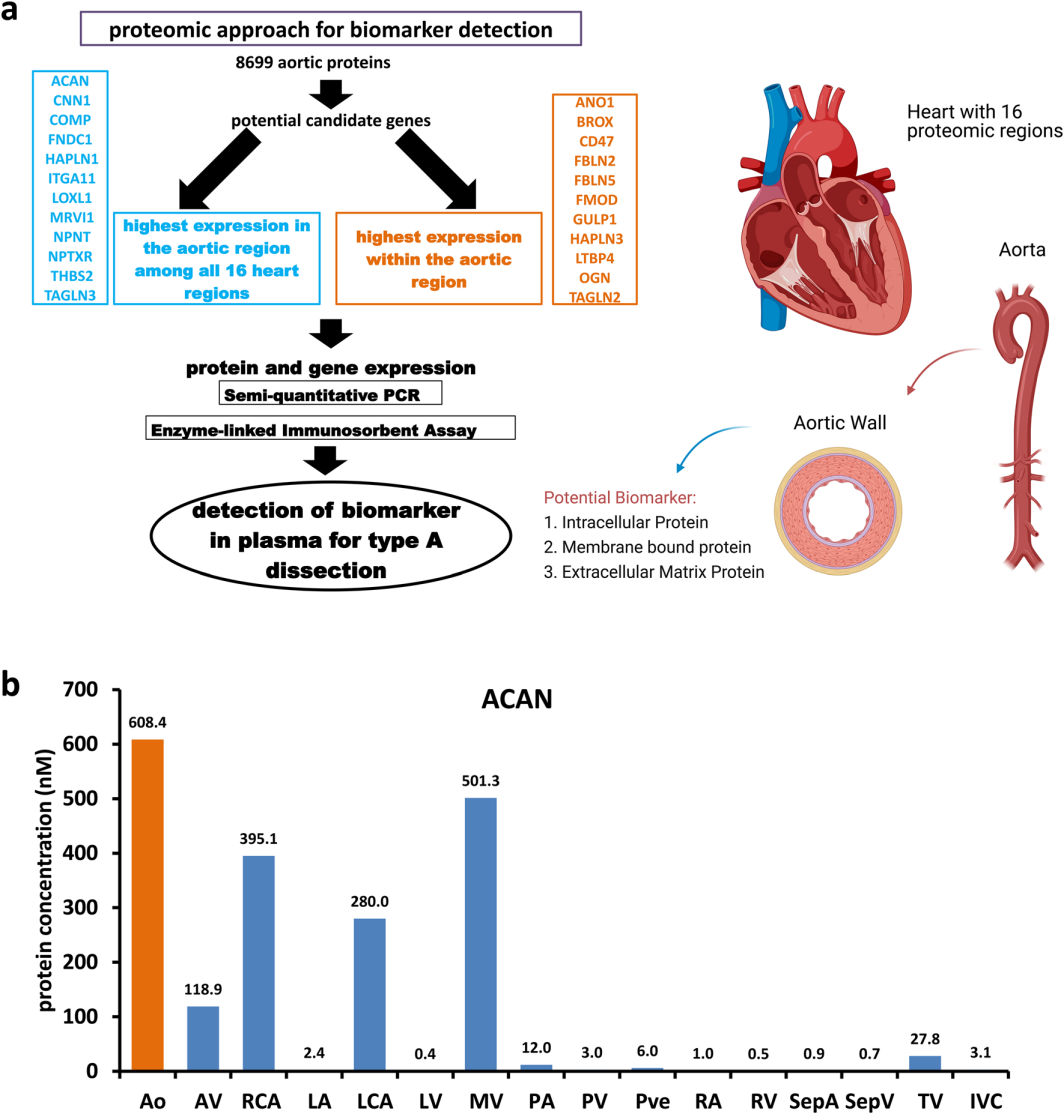
Workflow to identify candidate markers for acute type A aortic dissection patients and expression of selected candidate genes in human heart regions and surgical biopsies. **(a)** Strategy for selection and measurement of candidate biomarkers. **(b)** Protein expression of ACAN in different regions of the human heart. *Ao* aorta, *AV* aortic valve, *RCA* right coronary artery, *LA* left atrium, *LCA* left coronary artery, *LV* left ventricle, *MV* mitral valve, *PA* pulmonary artery, *PV* pulmonary valve, *Pve* pulmonary vein, *RA* right atrium, *RV* right ventricle, *SepA* atrial septum, *SepV* ventricular septum, *TV* tricuspid valve, *IVC* *inferior vena cava*.

ATAAD leads to a complete dissection of the physiological structure of the aortic wall^15^ and may thus induce release of proteins into the circulation. In our previous work we have identified 8699 proteins in the human aorta^16^. To limit the number of these possible candidates we conducted two approaches. Firstly, we selected proteins which were expressed most abundantly, but not necessarily restricted to aortic tissue. Secondly, we selected proteins which were preferentially expressed in aortic tissue compared to all other fifteen heart regions. Following these pre-selection criteria, we defined a list of 23 potential candidates (Fig. 1a). Looking at the protein expression of the candidate markers across the sixteen regions of the human heart, several candidates showed high expression in the aorta and coronary arteries (Supplementary Fig. S1), suggesting them as promising markers for vasculature. The concentration of ACAN protein, one promising candidate with a high specificity for arterial vessels across all sixteen regions of the human heart is shown in Fig. 1b.

**Figure 2:**
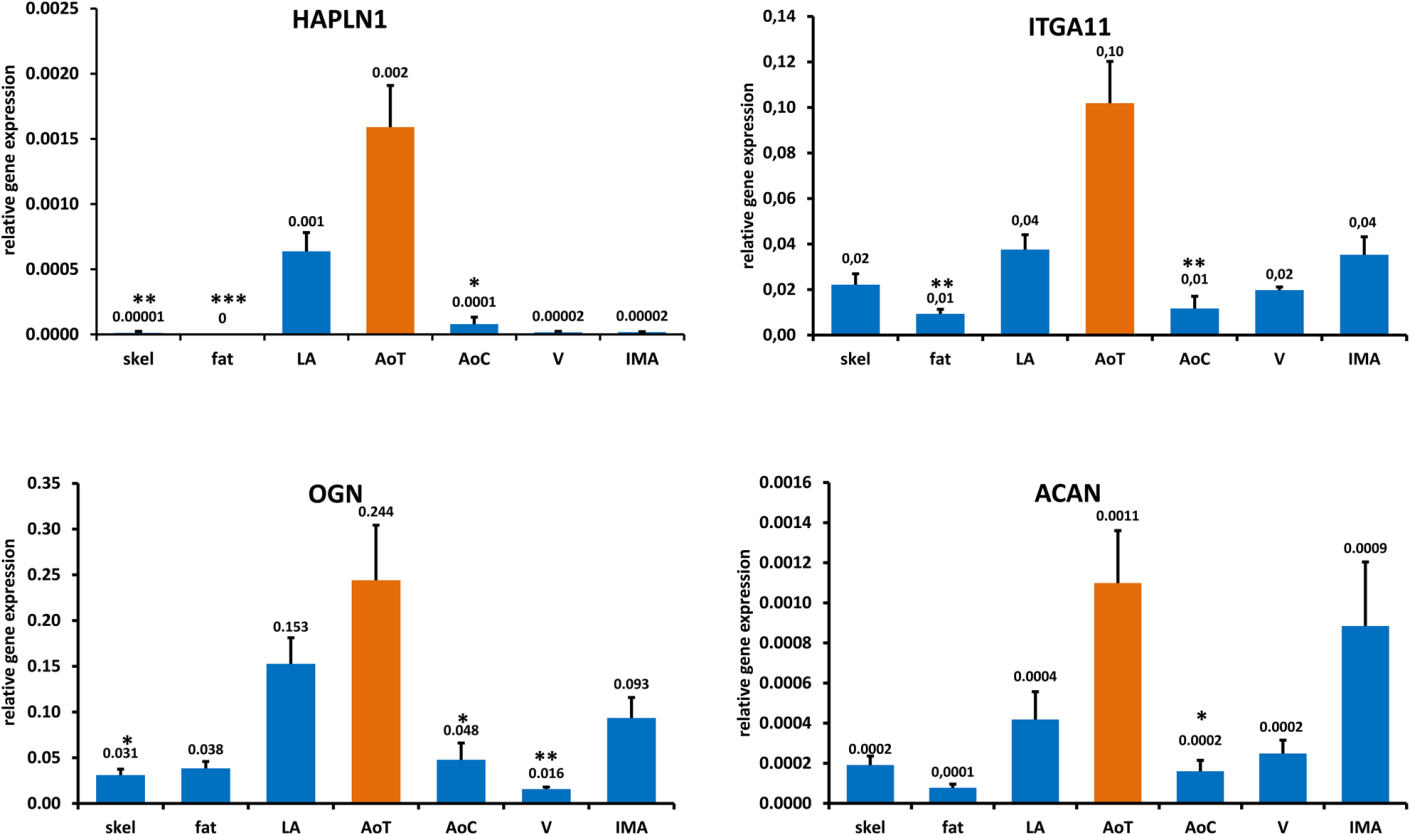
Gene expression in different surgical biopsies. Skel: skeletal muscle (n = 5), fat: subcutaneous fat (n = 5), LA: left atrium (n = 5), AoT: aorta from type A dissection (n = 6), AoC: aorta from coronary artery bypass graft (n = 6), V: *vena saphena magna* (n = 5), IMA: *arteria mammaria interna* (n = 5). Values represent means ± SEM. * *p* < 0.05, ** *p* < 0.01, *** *p* < 0.001 compared to aortic tissue from type A dissection. Significance of difference was tested with one-way ANOVA test.

Next, we determined mRNA expression of all candidates in aortic tissue from ATAAD patients in comparison to aortic tissue from coronary artery bypass patients. Furthermore, we measured mRNA in left atrial tissue, venous and arterial vessels and we analyzed the expression in extra-cardiac tissues such as fat and skeletal muscle. Figure 2 shows the expression of four candidate genes: *HAPLN1* (hyaluronan and proteoglycan link protein 1), *ITGA11* (integrin α-11), *OGN* (osteoglycin) and *ACAN* (aggrecan). In all cases the mRNA abundance is highest in the aorta from ATAAD patients and it is significantly different to aortic tissue from coronary artery bypass graft patients (Fig. 2). The mRNA expression of the remaining 19 candidates in these tissues is shown in Supplementary Fig. S2.

### ACAN protein concentration is enhanced in plasma of patients with acute type A aortic dissection

**Figure 3:**
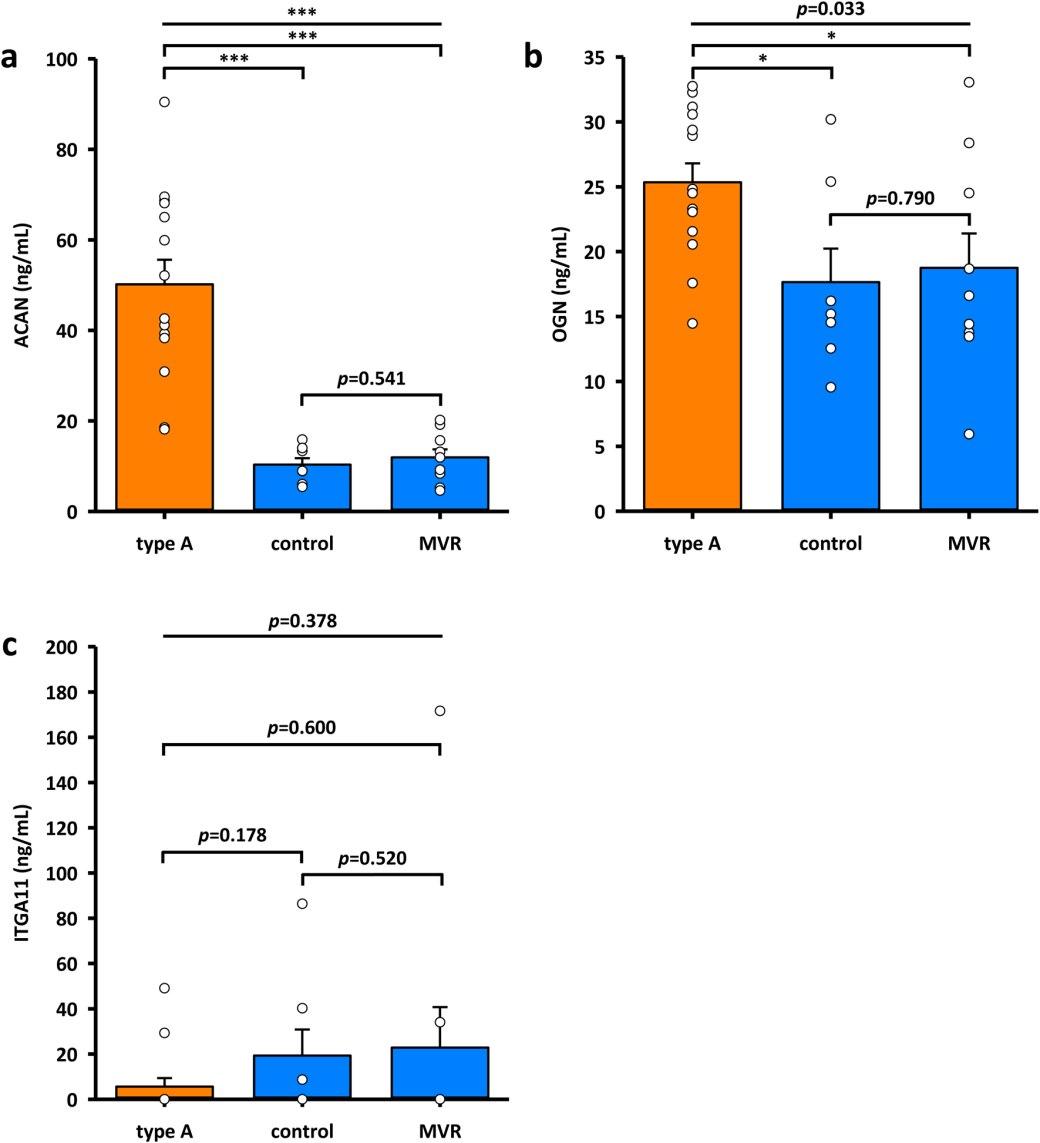
Protein concentration of candidate genes in plasma samples of type A dissection patients and control groups. Protein levels were quantified by commercial sandwich ELISA kits for ACAN [Aggrecan] **(a)**, OGN [Osteoglycin] **(b)** and ITGA11 [Integrin α 11] **(c)**. type A: acute type A aortic dissection (n = 14), control: healthy volunteers (n = 7), *MVR* mitral valve repair (n = 9). Values represent means ± SEM. ***p < 0.001. Significance of difference was tested with Wilcoxon-Mann–Whitney test.

Our data on protein and gene expression prompted us to determine the protein concentration of ACAN, OGN and ITGA11 in plasma samples of ATAAD patients, obtained directly after the arrival at our hospital. For comparison, we analyzed plasma of healthy volunteers and patients who underwent minimally invasive, isolated mitral valve repair (MVR). Indeed, ACAN levels were significantly elevated, with a four to five-fold higher concentration compared to both control groups (Fig. 3a). Mean plasma ACAN level was 50.16 ± 5.43 ng/mL. Mean plasma levels of the healthy subjects (control) and MVR group were 10.33 ± 1.42 ng/mL and 11.92 ± 1.77 ng/mL, respectively. The levels of OGN were also significantly enhanced in ATAAD samples with a mean value of 25.34 ± 1.46 ng/mL compared to control and MVR samples with 17.65 ± 2.58 ng/mL and 18.75 ± 2.65 ng/mL, respectively. However, the difference between ATAAD patients and control groups was much smaller (Fig. 3b). In contrast, ITGA11 values in plasma samples were lowest in the ATAAD group with 5.59 ± 3.79 ng/mL and similar in the two reference groups (control and MVR) with 19.31 ± 11.54 ng/µL and 22.84 ± 17.88 ng/mL (Fig. 3c).

### ACAN plasma levels are not enhanced in patients with acute myocardial infarction and aneurysm

**Figure 4:**
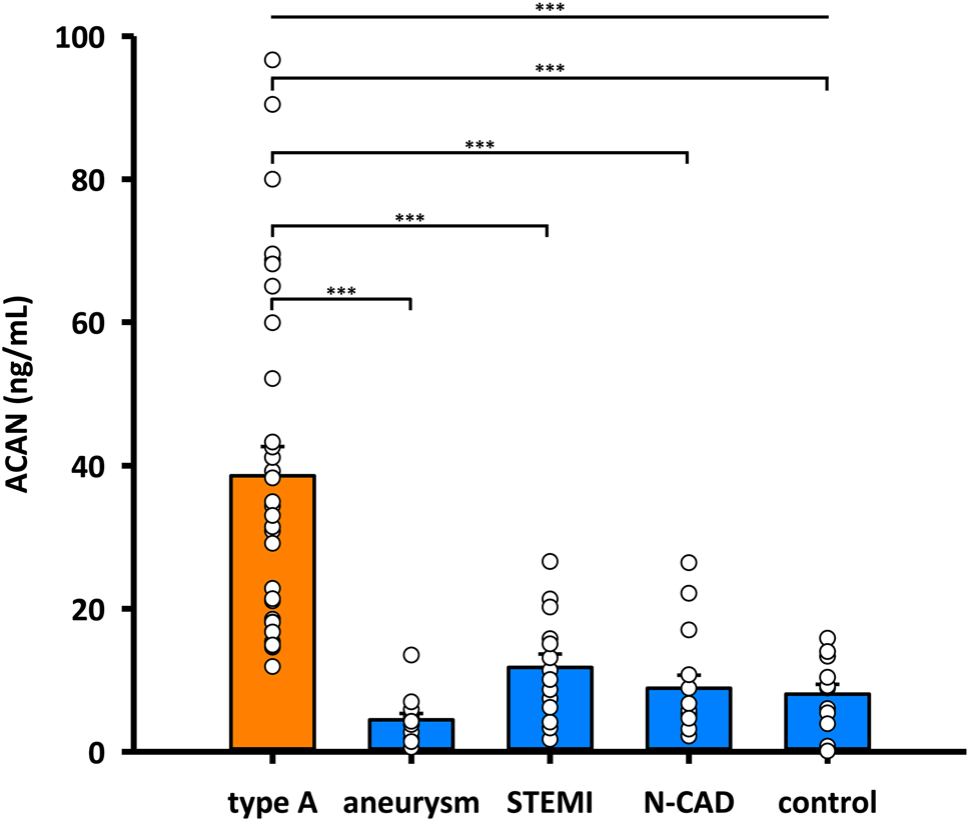
ACAN levels are not enhanced in plasma samples of patients without ATAAD. ACAN concentration in plasma of patients with ATAAD (type A, n = 33), asymptomatic chronic aneurysm of the ascending aorta (aneurysm, n = 13), MI with an acute ST-elevation (STEMI, n = 18), without known coronary artery disease (N-CAD, n = 15) and healthy volunteers (control, n = 12). Values represent means ± SEM. ***p < 0.001. Significance of difference was tested with Wilcoxon-Mann–Whitney test.

Next, we addressed the question whether elevated ACAN plasma levels are specific for ATAAD. To further substantiate our initial promising results, we increased the number of ATAAD patients (n = 33). Using this extended cohort, we detected a significant almost tenfold increase in plasma levels of ACAN in ATAAD patients with a mean plasma level of 38.59 ± 4.08 ng/mL compared to samples from patients with asymptomatic chronic aneurysm of the ascending aorta with a mean value of 4.45 ± 0.90 ng/mL (Fig. 4). We next analyzed ACAN plasma levels of patients with acute ST-elevation myocardial infarction (STEMI), which may confound the correct diagnosis of ATAAD. Again, ACAN protein concentrations of ATAAD patients were clearly and significantly elevated compared to STEMI patients who showed a mean value of 11.77 ± 1.89 ng/mL (Fig. 4). In addition, ACAN protein levels in patients without coronary artery disease (N-CAD) are significantly lower compared to ATAAD patients, but not significantly different to healthy controls or STEMI patients. N-CAD group showed a mean value of 8.88 ± 1.8 ng/mL (Fig. 4). Mean value of the healthy control group was 8.05 ± 1.38 ng/mL. The individual levels of CK-MB and cTnT and their correlation with ACAN for STEMI and N-CAD patients are shown in Supplementary Fig. S3. Thus, ACAN protein levels of ATAAD patients in the circulation are significantly elevated compared to healthy controls and patients with important cardiac differential diagnoses, including MI.

### Association of ACAN plasma concentration with demographic parameters and severity of ATAAD

**Figure 5:**
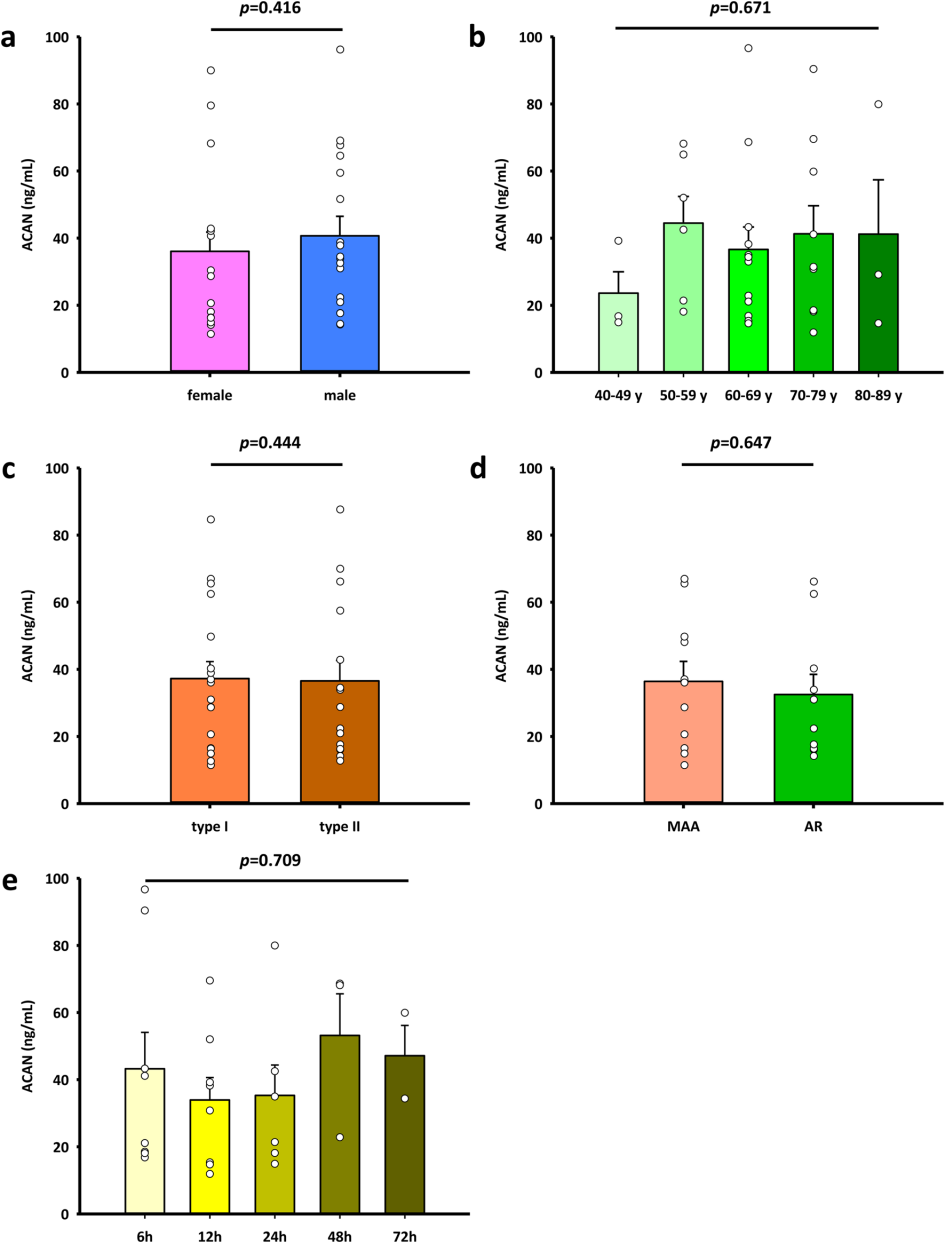
ACAN levels in ATAAD patients are not influenced by basic demographic parameters or the course of ATAAD. **(a)** ACAN levels in female (n = 15) and male (n = 18) ATAAD patients. **(b)** ACAN levels in ATAAD patients of different age (40–49 y, n = 3; 50–59 y, n = 6; 60–69 y, n = 12; 70–79 y, n = 9; ≥ 80 y, n = 3). **(c)** ACAN levels in DeBakey type I (*n* = 18) or type II (n = 15). **(d)** Correlation of ACAN levels and site of intimal tear. MAA (n = 11), AR (n = 10). **(e)** ACAN levels at 6 (n = 8), 12 (n = 8), 24 (n = 6), 48 (n = 3) or 72 h (n = 2) after the onset of the disease. *MAA* mid-ascending aorta, *AR* aortic root. Values are presented as means ± SEM. Significance of difference was tested with Wilcoxon-Mann–Whitney test or one-way ANOVA test.

Next, we addressed the question whether the release of ACAN into the circulation might be affected by basic demographic parameters such as sex or age. However, neither sex nor age had a significant impact on ACAN plasma levels (Fig. 5a,b). Mean ACAN plasma levels of female and male samples were 36.06 ± 5.74 ng/mL and 40.69 ± 5.80 ng/mL. For age association, the ATAAD samples were divided into five age groups. Mean ACAN plasma levels of the five age groups, organized from young to old, were 23.60 ± 6.38 ng/mL, 44.49 ± 7.94 ng/mL, 36.63 ± 6.69 ng/mL, 41.28 ± 8.37 ng/mL and 41.21 ng/mL (Fig. 5b). Despite the considerably lower mean ACAN level of 23.60 ng/mL in the first group (40–49 years) compared to the mean ACAN levels of the other four age groups, there was no statistically significant difference of ACAN plasma levels between all five groups according to the one-way-ANOVA test with a *p* value of 0.671. Furthermore, we considered whether extent of ATAAD, according to the De Bakey classification might be reflected by the ACAN concentration in plasma. However, there was no major difference between patients with De Bakey type I and II ATAAD (Fig. 5c) with mean ACAN levels of 37.24 ± 5.05 ng/mL and 36.53 ± 6.11 ng/mL. Next, we focused on the site of the intimal tear. Therefore, we compared ATAAD patients with an intimal tear in the aortic root (n = 10) with patients having an intimal tear in the mid ascending aorta or the aortic arch (n = 11). Mean serum ACAN levels were 32.5 ± 6.03 and 36.4 ± 5.94 ng/mL respectively (Fig. 5d, p = 0.647). Finally, we established kinetics of ACAN levels and the time period between onset of symptoms of ATAAD and the drawing of the blood samples. Shortest time period between symptom onset and blood withdrawal was 50 min due to an iatrogenic ATAAD during cardiac catheterization. In this patient measured ACAN level was 34.1 ng/mL. ACAN levels are also clearly elevated for up to 72 h after the onset without major differences at any time point. ACAN plasma levels at 6 h, 12 h, 24 h, 48 h and 72 h were 43.2 ± 10.84 ng/mL, 34.0 ± 6.67 ng/mL, 35.3 ± 9.06 ng/mL, 53.2 ± 12.39 ng/mL and 47.1 ± 9.03 ng/mL, respectively (*p* = 0.709, Fig. 5e).

### ACAN detects acute type A aortic dissection with high specificity and sensitivity

**Figure 6:**
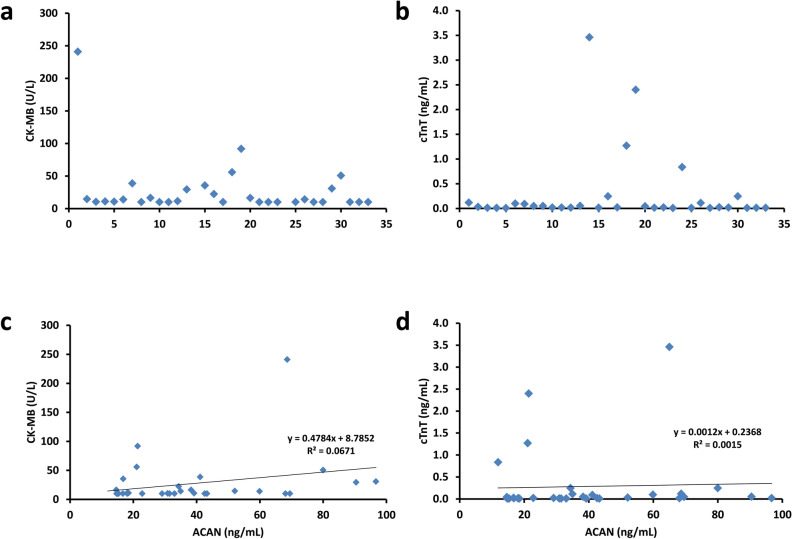
ACAN levels do not correlate with CK-MB or cTnT concentrations. **(a)** CK-MB levels of individual ATAAD patients. **(b)** Correlation between ACAN and CK-MB levels. **(c)** cTnT levels of individual ATAAD patients. **(d)** Correlation between ACAN and cTnT levels. *ACAN* aggrecan, CK-MB: creatine kinase-muscle brain isoform, *cTnT* cardiac troponin T, *STEMI* ST-elevation-myocardial-infarction.

**Figure 7:**
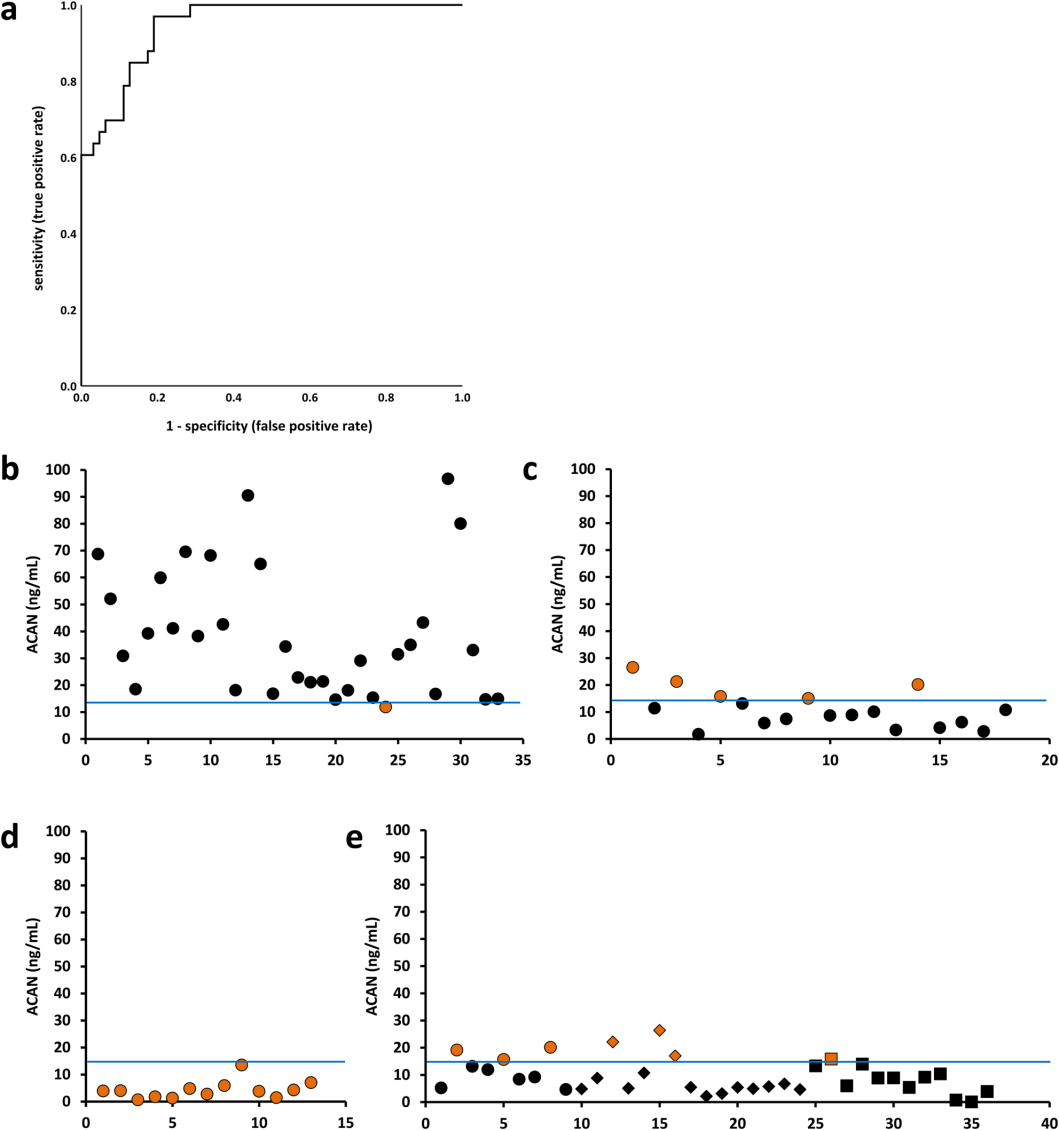
Sensitivity and specificity of ACAN to detect ATAAD. **(a)** Receiver-operating characteristics curve for all patients with ATAAD (n = 33) vs. all control subjects (n = 63). **(b)** ACAN levels in ATAAD patients (n = 33). **(c)** ACAN levels in STEMI patients (n = 18). **(d)** ACAN levels in patients with aneurysm (n = 13). **(E)** ACAN levels in surgical controls (MVR) (filled circle, n = 9), cardiac controls (N-CAD) (filled diamond, n = 15) and healthy persons (filled rectangle, n = 12). The blue line refers to the optimum discrimination level determined by the ROC analysis. Wrongly grouped samples are indicated in orange. *ACAN* Aggrecan, *ATAAD* acute thoracic aortic dissection, *MVR* mitral valve repair, *N-CAD* no coronary artery disease, *ROC* receiver-operator-curve, *STEMI* ST-elevation-myocardial-infarction.

We next evaluated the level of the already established clinical MI biomarkers, CK-MB and cTnT, in plasma samples of patients with ATAAD. For both markers, in the majority of samples, the values remained below the established clinical reference limit which defines myocardial cell damage (Fig. 6a,c). In addition, no correlation between plasma levels of ACAN and CK-MB (Fig. 6b) or cTnT (Fig. 6d) was seen. Area under the curve on receiver-operator characteristics (ROC) curve analysis for all ATAAD patients (n = 33) versus all control subjects (n = 63) was 0.947 (Fig. 7a). Based on the ROC curve analysis an ACAN concentration of 14.3 ng/mL in the plasma was the optimum discrimination limit, resulting in a sensitivity of 97% and a specificity of 81%. Chi-square analysis showed a positive predictive value of 72.7% and a negative predictive value of 98%. Only one ATAAD sample showed an ACAN concentration below this threshold (Fig. 7b). Analyzing the ACAN levels in patients with cardiac complications (STEMI or aneurysms, Fig. 7c,d) showed a specificity of more than 80%. In addition, in different experimental control groups (Fig. 7e) a similar specificity was obtained.

## Discussion

ATAAD is a very severe cardiovascular diagnosis with an emergency department incidence of 5.93/100.000 to 24.92/100,000^17^. ATAAD patients are often hospitalized with concomitant co-morbidities which mask and complicate the diagnosis of ATAAD^5^, demanding a high specificity next to a high sensitivity for a reliable biomarker. In our study we have measured ACAN levels in peripheral blood of ATAAD patients. Our data clearly show that ACAN concentrations were significantly increased in plasma of ATAAD patients compared to plasma samples of healthy individuals and patients suffering from different cardiovascular disease.

ACAN levels in ATAAD patients were elevated above our calculated threshold of 14.3 ng/mL based on the ROC curve analysis. In contrast, the ACAN plasma levels of the majority of patients with MI remained below this value. A minority of patients with elevated cardiac enzyme levels above the established clinical threshold most probably suffered from involvement of the aortic root with presumably consecutive narrowing or obstruction of the coronary ostia. Thus, the increase of ACAN in the peripheral circulation of ATAAD patients apparently happens completely independent of both CK-MB and cTnT.

Next, we analyzed a possible relationship between the extension of an acute dissection and the corresponding ACAN serum levels. Preoperative CT-scan data of all 33 ATAAD patients included in our study suggested a patent false lumen without partial or complete thrombosis (Table 1). These results were confirmed by intraoperative findings in each ATAAD patient. In our study we surprisingly observed no statistically significant difference in the preoperative ACAN serum levels of De Bakey Class I and II ATAAD patients. A possible explanation for this finding could be the difference in ACAN accumulation over the complete length of the aorta with a preferable accumulation within the ascending aorta compared to the descending part. Therefore, future studies should focus on the comparison of ACAN levels between type A and type B dissections to rule out possible differences between ACAN levels and corresponding affected parts of the aorta.

Additionally, we analyzed the potential influence of the anatomical site of the intimal tear in ATAAD patients since the embryonic origin of the aortic root differs from the origin of the mid-ascending aortic tissue. However, our results revealed no statistically significant difference between ACAN serum levels of ATAAD patients with or without aortic root involvement.

In addition, aneurysmatic alterations of the ascending aorta without dissection also did not result in increased ACAN plasma levels. Therefore, our study could definitely rule out ACAN as a possible screening marker for aneurysmatic thoracic aortic disease without acute dissection. In summary, it can therefore be concluded that secondary cardiovascular diagnoses did not influence the level of ACAN in plasma for specific diagnosis of ATAAD.

Basic demographic parameters such as age and sex as well as the previously discussed extent of the ATAAD do not influence peripheral ACAN levels, suggesting that only the traumatic event of ATAAD would lead to ACAN release.

The next question we addressed was whether ACAN is a feasible biomarker for early diagnosis. It is well known that serum cardiac enzyme levels, i.e., CK-MB and hs-cTnT, only rise significantly up to four hours after cardiac injury^4^. The earliest admissions at our department for ATAAD were 50 and 90 min after the onset of symptoms respectively. The according preoperative serum ACAN levels were 34.1 and 85.1 ng/ml respectively and by extension considerable higher than our calculated threshold of 14.3 ng/ml. Hence, ACAN seems to be a suitable biomarker for early diagnosis of an ATAAD.

Applying the optimum discrimination limit of 14.3 ng/mL, based on the ROC curve analyses, across our cohort of ATAAD patients, healthy probands and patients with other cardiovascular diagnoses (MVR, N-CAD) yielded a specificity of more than 97% and a sensitivity of 81% when considering all of our experimental control groups. In addition, chi-square analysis revealed a positive predictive value of 72.7% and a negative predictive value of 98%. Even if we focused on clinical patients and excluded the healthy persons, we still ended up with a sensitivity of > 81%.

Calponin and D-dimer have been proposed as diagnostic tools in ATAAD^10,12^. D-dimer analysis for instance is currently recommended in patients with acute thoracic chest pain in combination with cardiac enzymes according to the 2014 ESC guidelines^18^. As diagnostic biomarker for ATAAD D-dimers showed a sensitivity of 97% with a specificity of 56%, a positive predictive value of 60% and a negative predictive value of 96% (CI 95%)^19^. Comparing our results with these two markers we found a superior specificity of ACAN to discriminate ATAAD and MI (≈ 73%). Importantly, ACAN levels did not correlate with CK-MB or cTnT concentrations. Thus, a combination of ACAN with these markers might be beneficial to further increase the sensitivity. Therefore, the combined use of ATAAD and MI markers in an emergency setting should prompt the treating physician to run the appropriate, more invasive, and time demanding diagnostic test for definitive confirmation. Thus, therapeutic delays could be reduced or even prevented. The level of calponin increases in ATAAD but decreases beyond 12 h after onset^10^. In contrast, upon arrival at the hospital ACAN level stayed elevated and did not vary substantially for up to 72 h after the onset of ATAAD. This might especially be crucial when ATAAD occurred before that time period.

Comparing the performance of ACAN with the existing markers like D-dimers and calponin^10,12^ clearly underlines the superiority of ACAN. Still, the values for specificity and sensitivity do not completely reach the confidence of the well-established high-sensitive troponin assay for detection of MI^20^.

Some limitations of our study must be mentioned. ACAN can make up a substantial part of cartilage and ACAN degradation is an important feature of osteoarthritis^21^. Though we can definitely exclude osteoarthritis in our patient cohort, it must be mentioned that this diagnosis could possibly confound the proper detection of ATAAD.

In summary, we have identified ACAN plasma levels as a reliable biomarker to detect the presence of an ATAAD. This marker reliably detected ATAAD patients in a very sensitive manner. At the same time, the biomarker showed a satisfying specificity which was not confounded by the presence of MI.

## Supplementary Information

Supplementary Information.

## Data Availability

All data are presented in the text and the Supplementary Information.
