# Supplementary material for: Aggrecan: a new biomarker for acute type A aortic dissection
Source: Sci Rep. 2021 May 14;11:10371. doi: 10.1038/s41598-021-89653-y (PMC8121825; doi:10.1038/s41598-021-89653-y)
Supplement: Supplementary file 1 — Supplementary Information. [file 41598_2021_89653_MOESM1_ESM.pdf]

SUPPLEMENTARY INFORMATION

Aggrecan - a new biomarker for acute type A aortic dissection

Karl C. König, Harald Lahm, Martina Dreßen, Stefanie A. Doppler, Stefan Eichhorn, Nicole Beck, Kathrin Kraehschuetz, Sophia Doll, Stefan Holdenrieder, Adnan Kastrati, Rüdiger Lange, Markus Krane[4]

Supplementary Table S1. List of patients and control persons

Supplementary Table S2. Tissues used for candidate gene expression analyses

Supplementary Table S3. Primers used for qRT-PCR analyses

Supplementary Figure S1. Protein expression of 22 candidate genes in different regions of the human heart.

Supplementary Figure S2. Gene expression of candidate genes in different surgical biopsies.

Supplementary Figure S3. ACAN serum protein level correlation with CK-MB or cTnT concentrations in patients with STEMI

Supplementary Table S1. List of patients and control persons

ID	sex	age [1]	type of OP	diagnosis
A6387	male	48	MVR	mitral valve insufficiency
A6443	male	33	MVR	mitral valve insufficiency
A6449	female	61	MVR	mitral valve insufficiency
A6477	male	66	MVR	mitral valve insufficiency
A6578	male	23	MVR	mitral valve insufficiency
A6596	male	66	MVR	mitral valve insufficiency
A6607	male	65	MVR	mitral valve insufficiency
A6622	male	50	MVR	mitral valve insufficiency
A6654	male	62	MVR	mitral valve insufficiency
	8 m/1 f	61 (23 – 66)		
A6543	female	74		aneurysm
A6751	female	62	Aorta	aneurysm
A6942	female	67		aneurysm
A7657	male	68	AVR + Aorta	aneurysm
A7691	male	54	Aorta	aneurysm
A7761	female	66	AVR + Aorta	aneurysm
A7767	male	71	AVR + Aorta	aneurysm
A7816	female	76	AVR + Aorta	aneurysm
A7857	male	55	AVR + Aorta	aneurysm
A7922	male	62	Aorta	aneurysm
A7939	female	80	TVR + Aorta	aneurysm
A8049	female	66	AVR + Aorta	aneurysm
	5 m/7 f	66.5 (54 – 80)		
A5898	female	62	AVR + Aorta	type A dissection
A6128	male	72	Aorta	type A dissection
A6286	male	64	CABG + AVR + Aorta	type A dissection
A6619	male	54	Aorta	type A dissection
A6767	female	70	Aorta	type A dissection
A6949	male	63		type A dissection
A7093	female	76	Aorta	type A dissection
A7095	male	46	AVR + Aorta	type A dissection
A7414	male	72	AVR + Aorta	type A dissection
A7486	female	74		type A dissection
A7502	male	52	AVR + Aorta	type A dissection
A7512	male	60	CABG + AVR + Aorta	type A dissection
A7687	female	51	AVR + Aorta	type A dissection
A7742	male	71	AVR + Aorta	type A dissection
A7917	female	78	Aorta	type A dissection
A7933	male	57		type A dissection
A8086	female	64	AVR + Aorta	type A dissection
A8170	female	64	Aorta	type A dissection
A8288	male	51	CABG + AVR + Aorta	type A dissection
A8303	female	85	Aorta	type A dissection
A8315	male	55	Aorta	type A dissection
A8363	female	88	AVR + Aorta	type A dissection
A8480	female	76	Aorta	type A dissection
A8482	female	65	Aorta	type A dissection
A8487	male	71	CABG + AVR + Aorta	type A dissection
A8488	male	65	AVR + Aorta	type A dissection
A8505	female	67	Aorta	type A dissection
A8645	female	43	CABG + AVR + Aorta	type A dissection
A8734	male	66	AVR + Aorta	type A dissection
A8934	female	82	AVR + Aorta	type A dissection

ID	sex	age [1]	type of OP	diagnosis
A8971	male	59	AVR + Aorta	type A dissection
A9075	male	64	Aorta	type A dissection
A9082	male	48	Aorta	type A dissection
	18 m/15 f	64 (43 – 88)		
558	female	77	none	STEMI
1244	female	68	none	STEMI
1278	male	67	none	STEMI
1297	male	64	none	STEMI
1415	male	58	none	STEMI
1465	female	76	none	STEMI
1520	male	65	none	STEMI
1521	female	47	none	STEMI
1572	male	58	none	STEMI
1621	female	56	none	STEMI
1643	male	67	none	STEMI
1716	female	54	none	STEMI
1800	female	75	none	STEMI
A5878	male	58	CABG	STEMI
	9 m/9 f	64 (54 – 77)		
289	male	52	none	non coronary artery disease
758	male	71	none	non coronary artery disease
814	male	59	none	non coronary artery disease
877	male	64	none	non coronary artery disease
1183	female	72	none	non coronary artery disease
1194	female	71	none	non coronary artery disease
1200	female	69	none	non coronary artery disease
1201	female	72	none	non coronary artery disease
1225	male	68	none	non coronary artery disease
1229	male	49	none	non coronary artery disease
1255	female	63	none	non coronary artery disease
1265	female	81	none	non coronary artery disease
1274	female	55	none	non coronary artery disease
1279	male	85	none	non coronary artery disease
1288	female	68	none	non coronary artery disease
	7 m/8 f	68 (49 – 85)		
A5480	female	39	none	none
A5481	female	27	none	none
A5482	female	32	none	none
A5484	male	60	none	none
A5485	male	41	none	none
A5486	female	26	none	none
A5489	female	39	none	none
A8213	male	47	none	none
A8214	female	52	none	none
A8215	female	33	none	none
A8216	female	34	none	none
A8217	male	40	none	none
	4 m/8 f	39 (26 – 60)		

[1] at the time of operation, CABG: coronary artery bypass graft, AVR: aortic valve replacement, MVR: mitral valve replacement, TVR: tricuspid valve replacement, STEMI: acute ST elevation myocardial infarction

Supplementary Table S2. Tissues used for candidate gene expression analyses

ID	sex	age [1]	tissue	type of OP	diagnosis
A5578	male	74	skeletal muscle	CABG	coronary heart disease
A5579	male	73	skeletal muscle	CABG	coronary heart disease
A5588	male	82	skeletal muscle	CABG	coronary heart disease
A5646	male	59	skeletal muscle	CABG	coronary heart disease
A5652	male	61	skeletal muscle	CABG	coronary heart disease
A5579	male	73	subcutaneous fat	CABG	coronary heart disease
A5588	male	82	subcutaneous fat	CABG	coronary heart disease
A5598	male	65	subcutaneous fat	CABG	coronary heart disease
A5607	male	63	subcutaneous fat	CABG + AVR	coronary heart disease
A5619	male	53	subcutaneous fat	CABG	coronary heart disease
A4991	male	75	left atrium	CABG	coronary heart disease
A4996	male	73	left atrium	MVP	coronary heart disease
A5013	male	71	left atrium	CABG	coronary heart disease
A5053	male	64	left atrium	CABG + MVR	coronary heart disease
A5065	male	73	left atrium	MVR + TVR	coronary heart disease
A3642	male	64	aorta	AVR + aorta	acute type dissection
A4952	male	44	aorta	AVR + aorta	acute type dissection
A5797	male	48	aorta	aorta	acute type dissection
A5898	male	62	aorta	AVR + aorta	acute type dissection
A6128	male	72	aorta	aorta	acute type dissection
A6767	female	70	aorta	aorta	acute type dissection
A3677	male	76	aorta	CABG	coronary heart disease
A4613	male	57	aorta	CABG	coronary heart disease
A4902	male	78	aorta	CABG	coronary heart disease
A5199	male	71	aorta	CABG	coronary heart disease
A5356	male	57	aorta	CABG	coronary heart disease
A5644	male	53	aorta	CABG	coronary heart disease
A623	male	62	vena saphena magna	CABG + AVR	coronary heart disease
A629	male	76	vena saphena magna	CABG	coronary heart disease
A5305	female	77	vena saphena magna	CABG	coronary heart disease
A5334	female	84	vena saphena magna	CABG	coronary heart disease
A5352	female	62	vena saphena magna	CABG + AVR + MVR + TVR	coronary heart disease
A296	female	69	arteria mammaria interna	CABG	coronary heart disease
A5141	male	62	arteria mammaria interna	CABG	coronary heart disease
A5142	male	75	arteria mammaria interna	CABG	coronary heart disease
A5199	male	71	arteria mammaria interna	CABG	coronary heart disease
A5216	female	47	arteria mammaria interna	CABG	coronary heart disease

[1] at the time of operation, CABG: coronary artery bypass graft, AVR: aortic valve repair, MVR: mitral valve repair, TVR: tricuspid valve repair

SupplementaryTable S3. Primers used for qRT-PCR analyses

primer	sequence	length of amplicon
hu b-actin_F382	5’ CCAACCGCGAGAAGATGA 3’	
hu b-actin_R478	5’ CCAGAGGCGTACAGGGATAG 3’	97 bp
hu ACAN_F2609	5’ CTATACCCCAGTGGGCACAT 3’	
hu ACAN_R2726	5’ GGCACTTCAGTTGCAGAAGG 3’	118 bp
hu ANO1_F1738	5’ GGCATATTCCAGAGGAGTCAA 3’	
hu ANO1_R1833	5’ TCCATGTCAGCTTCACTTTGTC 3’	95 bp
hu BROX_F474	5’ TAGTCACTGGCCCTTCTGCT 3’	
hu BROX_R567	5’ GGATTACAGCTCAAATCAGTGAAC 3’	94 bp
hu CD47_F558	5’ CATCGAGCTAAAATATCGTGTTG 3’	
hu CD47_R658	5’ ACTGTCCCCAGAACAGGAGTA 3’	101 bp
hu CNN1_F357	5’ ACTTCATGGACGGCCTCA 3’	
hu CNN1_R451	5’ TTGGGTTGACTCATTGATCTTC 3’	95 bp
hu COMP_F1683	5´ CAACTGGGTGGTGCTCAA 3´	
hu COMP_R1780	5´ AGTCCACGCCATTGAAGG 3´	98 bp
hu FBLN5_F430	5’ AGCTTTCTTCTCGCCTTCG 3’	
hu FBLN5_R520	5’ CAGAGAGCCAGAATGGTAAACAG 3’	91 bp
hu FMOD_F1310	5’ CAACCAGCTGCAGAAGATCC 3’	
hu FMOD_R1406	5’ GCAGAAGCTGCTGATGGAG 3’	97 bp
hu FNDC1_F4857	5’ CCTACGGAAGAGGCCTACG 3’	
hu FNDC1_R4941	5’ TGGTCGAAGGCTCAGTGG 3’	85 bp
hu GULP1_F645	5’ AACATTTATTTGGCTGATCCTCA 3’	
hu GULP1_R720	5’ AGGTGTATGCATCCATGTTTTG 3’	76 bp
hu HAPLN1_F201	5’ GAGGAGAAAGAGCGCTAGCTT 3’	
hu HAPLN1_R309	5’ AGCCCAAAGAATCTTCTTCACTT 3’	109 bp
hu HAPLN3_F783	5’ TTACCGCTGTGAGGTCATTG 3’	
hu HAPLN3_R873	5’ GGGGGACTGGTAAGGAAAGA 3’	91 bp
hu ITGA11_F3196	5’ TGGGGCAATAGCACTGAGTA 3’	
hu ITGA11_R3288	5’ GGAGACGACATCAGAGTTGCT 3’	93 bp
hu LOXL1_F1604	5’ GAAGAACCAGGGCACAGC 3’	
hu LOXL1_R1691	5’ CATGCTGTGGTAATGCTGGT 3’	88 bp
hu LTBP4_F1079	5’ CATTCTGCGGAACATCACTAAA 3’	
hu LTBP4_R1194	5’ GGCAGATCTCCCGGAAAC 3’	116 bp
hu MRVI1_F2059	5’ CTGAAAGGGAACGCAACCT 3’	
hu MRVI1_R2151	5’ GGTGCCAGAGTGAAGCTGA 3’	93 bp
hu NPNT_F750	5’ TGATGGGAGGACCTGTGTAGA 3’	
hu NPNT_R835	5’ CAAAAGTGTTGACACATTGCCTA 3’	107 bp
hu NPTXR_F941	5‘ CAGAGGCAGGAAGTGGAAAA 3’	
hu NPTXR_R1032	5‘ GCATCTGGAGGACTGTAGGC 3’	86 bp
hu OGN_F526	5’ CCTCCCAAGAAAGAAAATGATG 3’	
hu OGN_R634	5’ TTGGTAAGGGTGGTACAGCA 3’	109 bp
hu TAGLN2_F276	5’ TCCAGAACTGGCTCAAGGAT 3’	
hu TAGLN2_R396	5’ TGCTCCATCTGCTTGAAGG 3’	121 bp
hu TAGLN3_F945	5’ TTTAGGCAGCGTTGCAGTC 3’	
hu TAGLN3_R1038	5’ AAAGCCTCTCCGATTCTGCT 3’	94 bp
hu THBS2_F1183	5’ ACCAGCTCAGCGAGAACCT 3’	
hu THBS2_R1279	5’ CAAGCTGACATGTTCCTTGTCT 3’	97 bp

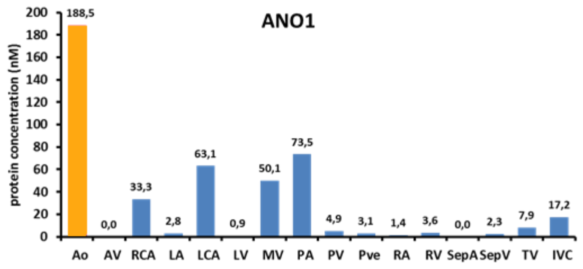
ANO1
protein concentration (nM)
188,5
0,0
33,3
2,8
63,1
0,9
50,1
73,5
4,9
3,1
1,4
3,6
0,0
2,3
7,9
17,2
Ao AV RCA LA LCA LV MV PA PV Pve RA RV SepA SepV TV IVC

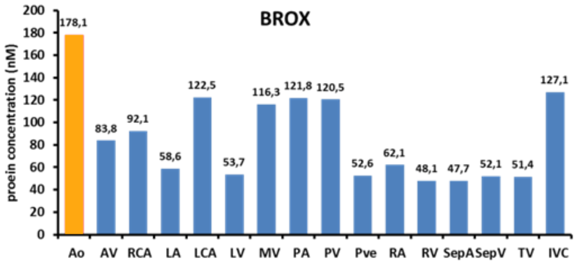
BROX
proein concentration (nM)
178,1
83,8
92,1
58,6
122,5
53,7
116,3
121,8
120,5
52,6
62,1
48,1
47,7
52,1
51,4
127,1
Ao AV RCA LA LCA LV MV PA PV Pve RA RV SepA SepV TV IVC

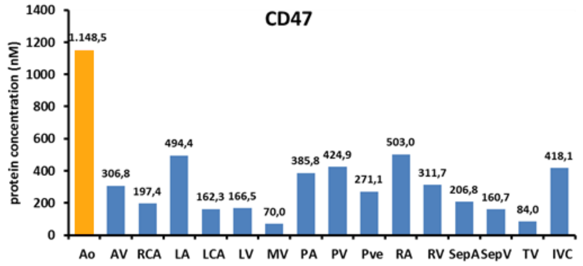
CD47
protein concentration (nM)
1.148,5
306,8
197,4
494,4
162,3
166,5
70,0
385,8
424,9
271,1
503,0
311,7
206,8
160,7
84,0
418,1
Ao AV RCA LA LCA LV MV PA PV Pve RA RV SepA SepV TV IVC

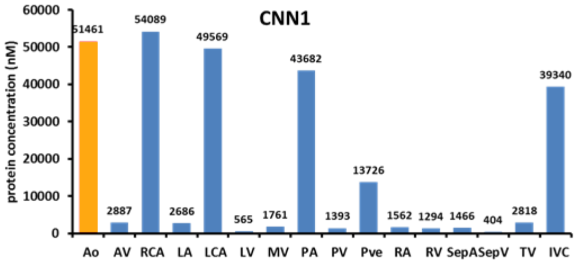
CNN1
protein concentration (nM)
51461
2887
54089
2686
49569
565
1761
43682
1393
13726
1562
1294
1466
404
2818
39340
Ao AV RCA LA LCA LV MV PA PV Pve RA RV SepA SepV TV IVC

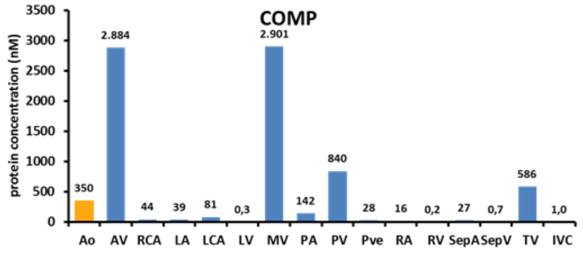
COMP
protein concentration (nM)
350
2.884
44
39
81
0,3
2.901
142
840
28
16
0,2
27
0,7
586
1,0
Ao AV RCA LA LCA LV MV PA PV Pve RA RV SepA SepV TV IVC

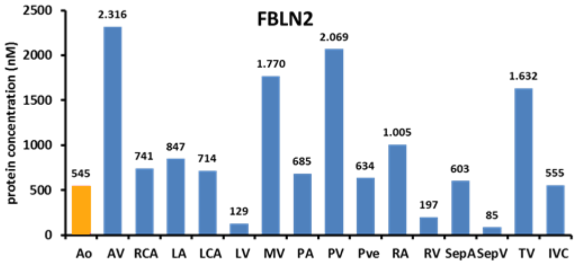
FBLN2
protein concentration (nM)
545
2.316
741
847
714
129
1.770
685
2.069
634
1.005
197
603
85
1.632
555
Ao AV RCA LA LCA LV MV PA PV Pve RA RV SepA SepV TV IVC

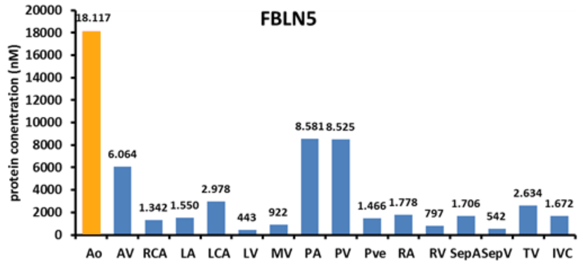
FBLN5
protein concentration (nM)
18.117
6.064
1.342
1.550
2.978
443
922
8.581
8.525
1.466
1.778
797
1.706
542
2.634
1.672
Ao AV RCA LA LCA LV MV PA PV Pve RA RV SepA SepV TV IVC

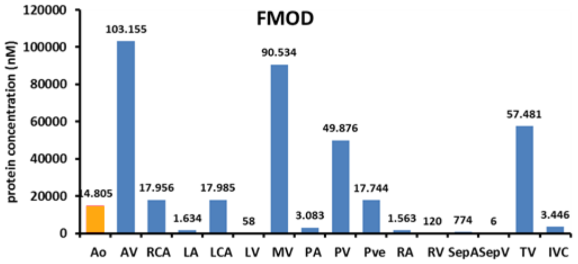
FMOD
protein concentration (nM)
14.805
103.155
17.956
1.634
17.985
58
90.534
3.083
49.876
17.744
1.563
120
774
6
57.481
3.446
Ao AV RCA LA LCA LV MV PA PV Pve RA RV SepA SepV TV IVC

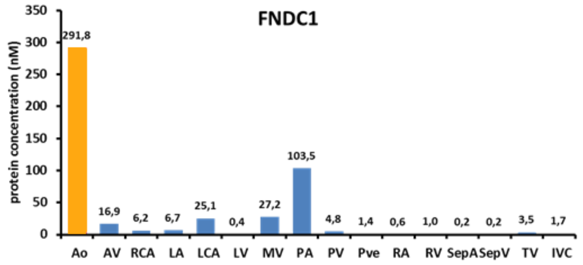
FNDC1
protein concentration (nM)
291,8
16,9
6,2
6,7
25,1
0,4
27,2
103,5
4,8
1,4
0,6
1,0
0,2
0,2
3,5
1,7
Ao AV RCA LA LCA LV MV PA PV Pve RA RV SepA SepV TV IVC

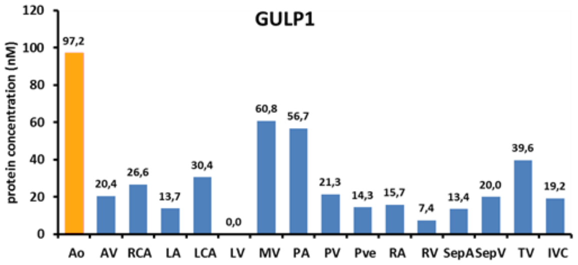
GULP1
protein concentration (nM)
97,2
20,4
26,6
13,7
30,4
0,0
60,8
56,7
21,3
14,3
15,7
7,4
13,4
20,0
39,6
19,2
Ao AV RCA LA LCA LV MV PA PV Pve RA RV SepA SepV TV IVC

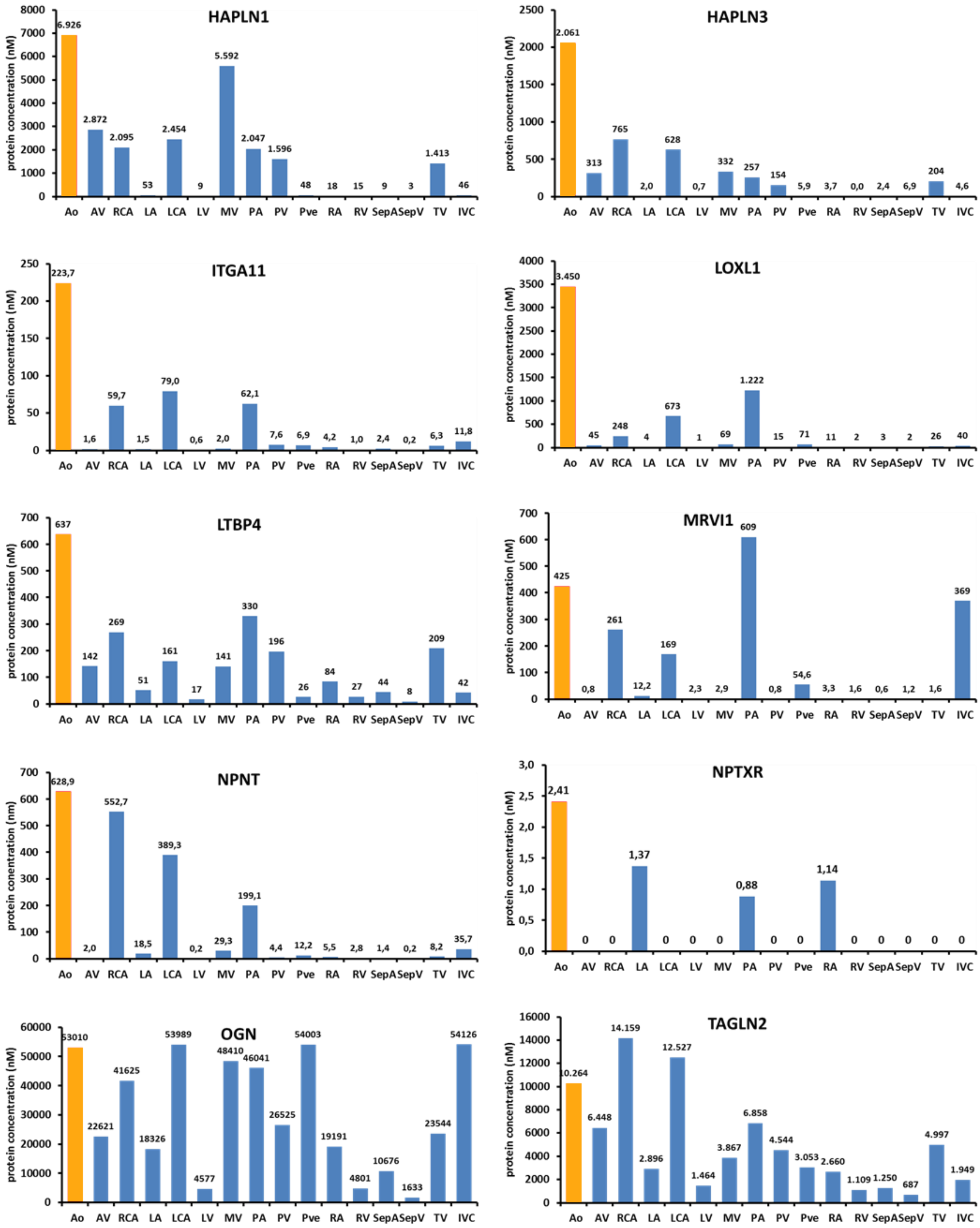
HAPLN1
protein concentration (nM)
6.926
2.872
2.095
53
2.454
9
5.592
2.047
1.596
48
18
15
9
3
1.413
46
HAPLN3
protein concentration (nM)
2.061
313
765
2,0
628
0,7
332
257
154
5,9
3,7
0,0
2,4
6,9
204
4,6
ITGA11
protein concentration (nM)
223,7
1,6
59,7
1,5
79,0
0,6
2,0
62,1
7,6
6,9
4,2
1,0
2,4
0,2
6,3
11,8
LOXL1
protein concentration (nM)
3.450
45
248
4
673
1
69
1.222
15
71
11
2
3
2
26
40
LTBP4
protein concentration (nM)
637
142
269
51
161
17
141
330
196
26
84
27
44
8
209
42
MRVI1
protein concentration (nM)
425
0,8
261
12,2
169
2,3
2,9
609
0,8
54,6
3,3
1,6
0,6
1,2
1,6
369
NPNT
protein conentration (nm)
628,9
2,0
552,7
18,5
389,3
0,2
29,3
199,1
4,4
12,2
5,5
2,8
1,4
0,2
8,2
35,7
NPTXR
protein concentration (nM)
2,41
0
0
1,37
0
0
0
0,88
0
0
1,14
0
0
0
0
0
OGN
protein concentration (nM)
53010
22621
41625
18326
53989
4577
48410
46041
26525
54003
19191
4801
10676
1633
23544
54126
TAGLN2
protein concentration (nM)
10.264
6.448
14.159
2.896
12.527
1.464
3.867
6.858
4.544
3.053
2.660
1.109
1.250
687
4.997
1.949
Ao AV RCA LA LCA LV MV PA PV Pve RA RV SepA SepV TV IVC

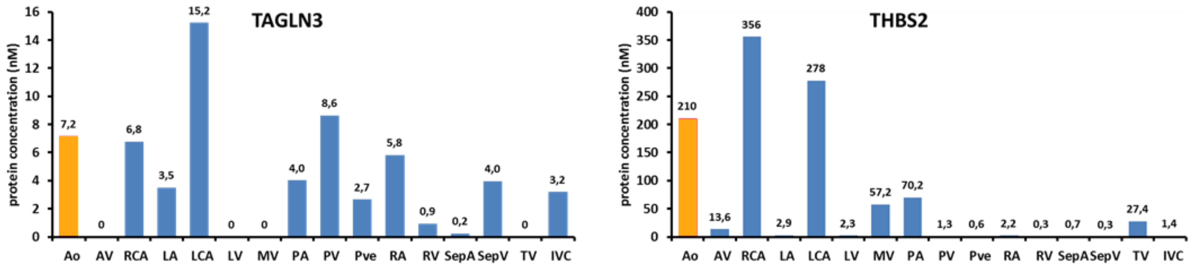

Supplementary Figure S1. Protein expression of 22 candidate genes in different regions of the human heart. Ao: aorta, AV: aortic valve, RCA: right coronary artery, LA: left atrium, LCA: left coronary artery, LV: left ventricle, MV: mitral valve, PA: pulmonary artery, PV: pulmonary valve, Pve: pulmonary vein, RA: right atrium, RV: right ventricle, SepA: atrial septum, SepV: ventricular septum, TV: tricuspid valve, IVC: inferior vena cava.

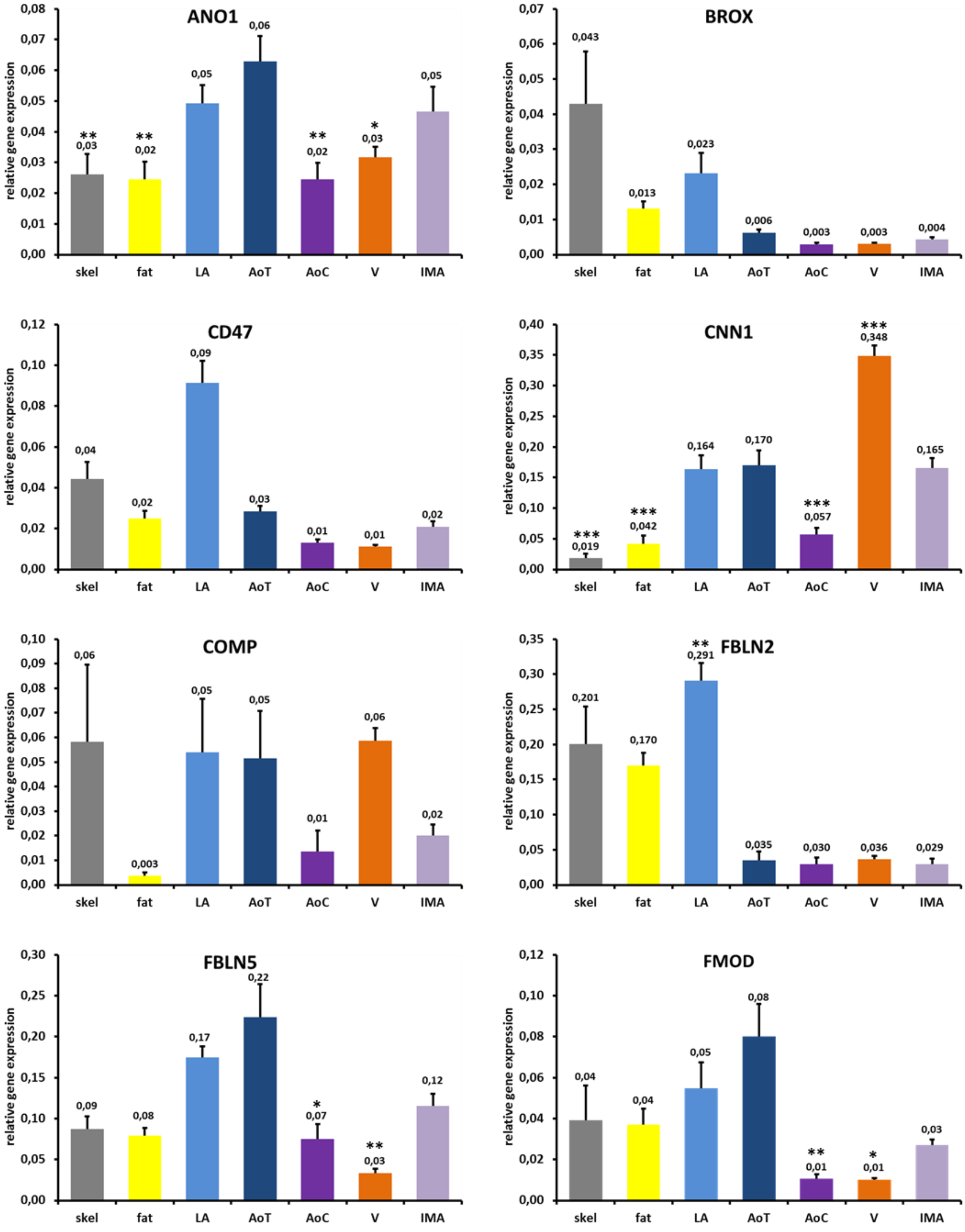
ANO1
relative gene expression
0,08
0,07
0,06
0,05
0,04
0,03
0,02
0,01
0,00
** 0,03
** 0,02
0,05
0,06
** 0,02
* 0,03
0,05
skel
fat
LA
AoT
AoC
V
IMA
BROX
relative gene expression
0,07
0,06
0,05
0,04
0,03
0,02
0,01
0,00
0,043
0,013
0,023
0,006
0,003
0,003
0,004
skel
fat
LA
AoT
AoC
V
IMA
CD47
relative gene expression
0,12
0,10
0,08
0,06
0,04
0,02
0,00
0,04
0,02
0,09
0,03
0,01
0,01
0,02
skel
fat
LA
AoT
AoC
V
IMA
CNN1
relative gene expression
0,40
0,35
0,30
0,25
0,20
0,15
0,10
0,05
0,00
*** 0,019
*** 0,042
0,164
0,170
*** 0,057
*** 0,348
0,165
skel
fat
LA
AoT
AoC
V
IMA
COMP
relative gene expression
0,10
0,09
0,08
0,07
0,06
0,05
0,04
0,03
0,02
0,01
0,00
0,06
0,003
0,05
0,05
0,01
0,06
0,02
skel
fat
LA
AoT
AoC
V
IMA
FBLN2
relative gene expression
0,35
0,30
0,25
0,20
0,15
0,10
0,05
0,00
0,201
0,170
** 0,291
0,035
0,030
0,036
0,029
skel
fat
LA
AoT
AoC
V
IMA
FBLN5
relative gene expression
0,30
0,25
0,20
0,15
0,10
0,05
0,00
0,09
0,08
0,17
0,22
* 0,07
** 0,03
0,12
skel
fat
LA
AoT
AoC
V
IMA
FMOD
relative gene expression
0,12
0,10
0,08
0,06
0,04
0,02
0,00
0,04
0,04
0,05
0,08
** 0,01
* 0,01
0,03
skel
fat
LA
AoT
AoC
V
IMA

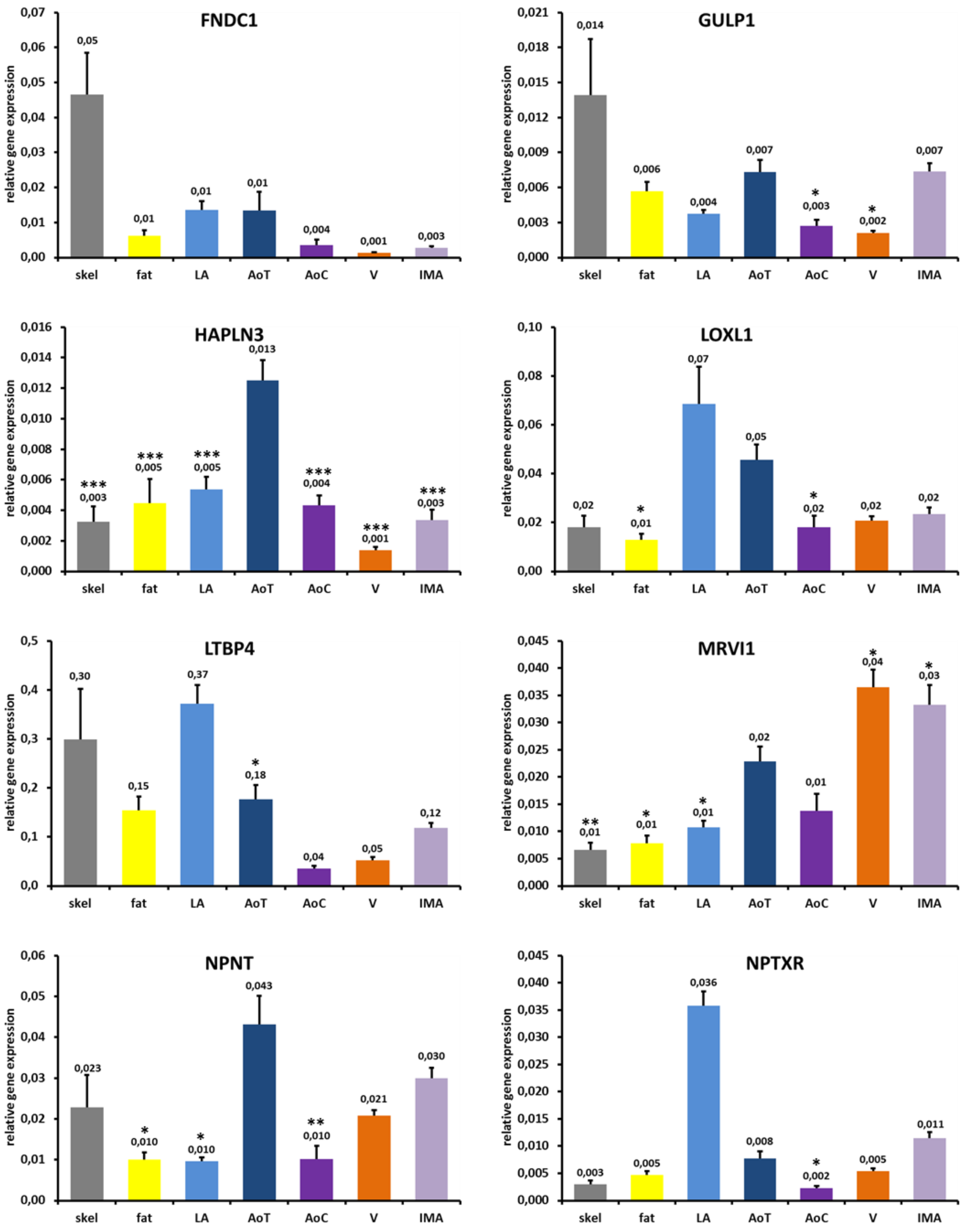
FNDC1
relative gene expression
0,07
0,06
0,05
0,04
0,03
0,02
0,01
0,00
0,05
0,01
0,01
0,01
0,004
0,001
0,003
skel
fat
LA
AoT
AoC
V
IMA
GULP1
0,021
0,018
0,015
0,012
0,009
0,006
0,003
0,000
0,014
0,006
0,004
0,007
*
0,003
*
0,002
0,007
HAPLN3
0,016
0,014
0,012
0,010
0,008
0,006
0,004
0,002
0,000
***
0,003
***
0,005
***
0,005
0,013
***
0,004
***
0,001
***
0,003
LOXL1
0,10
0,08
0,06
0,04
0,02
0,00
0,02
*
0,01
0,07
0,05
*
0,02
0,02
0,02
LTBP4
0,5
0,4
0,3
0,2
0,1
0,0
0,30
0,15
0,37
*
0,18
0,04
0,05
0,12
MRVI1
0,045
0,040
0,035
0,030
0,025
0,020
0,015
0,010
0,005
0,000
**
0,01
*
0,01
*
0,01
0,02
0,01
*
0,04
*
0,03
NPNT
0,06
0,05
0,04
0,03
0,02
0,01
0,00
0,023
*
0,010
*
0,010
0,043
**
0,010
0,021
0,030
NPTXR
0,045
0,040
0,035
0,030
0,025
0,020
0,015
0,010
0,005
0,000
0,003
0,005
0,036
0,008
*
0,002
0,005
0,011

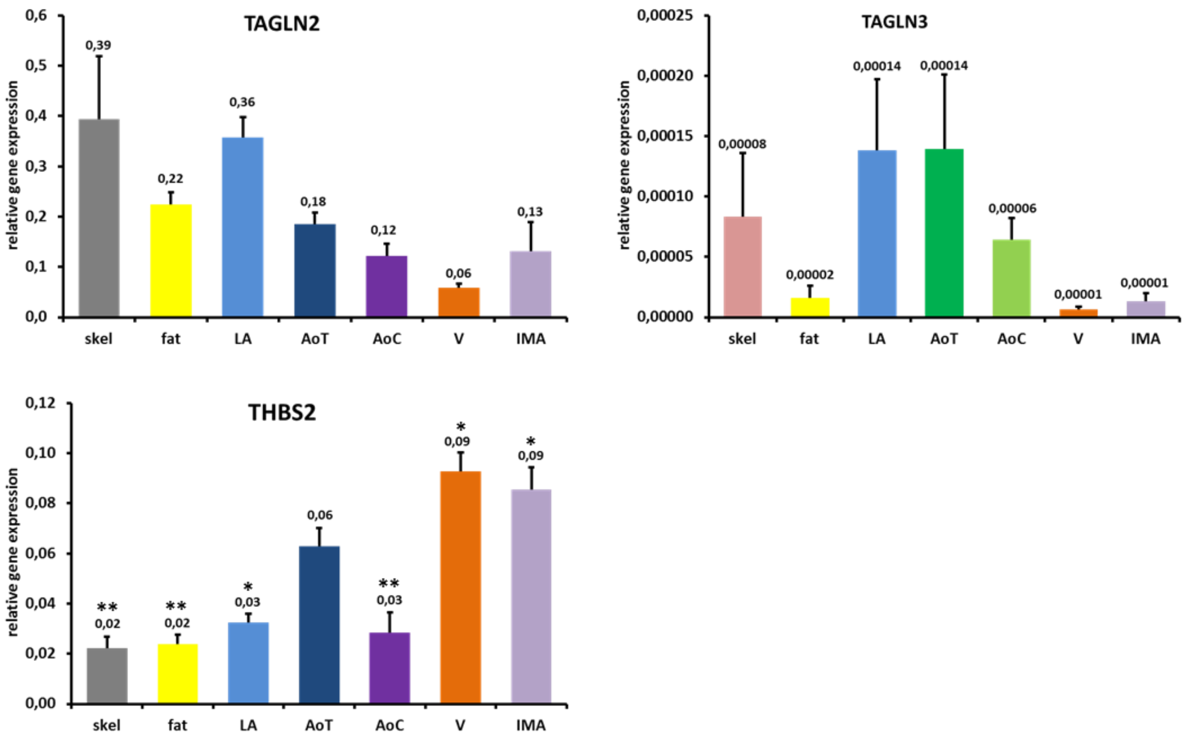

Supplementary Figure S2. Gene expression of candidate genes in different surgical biopsies. Skel: skeletal muscle (n=5), fat: subcutaneous fat (n=5), LA: left atrium (n=5), AoT: aorta from type A dissection (n=6), AoC: aorta from coronary artery bypass graft (n=6), V: vena saphena magna (n=5), IMA: arteria mammaria interna (n=5). Values represent means ± SE. * $p < 0.05$, ** $p < 0.01$ compared to aortic tissue from type A dissection. Significance of difference was tested with one way ANOVA or the Kruskal-Wallis test.

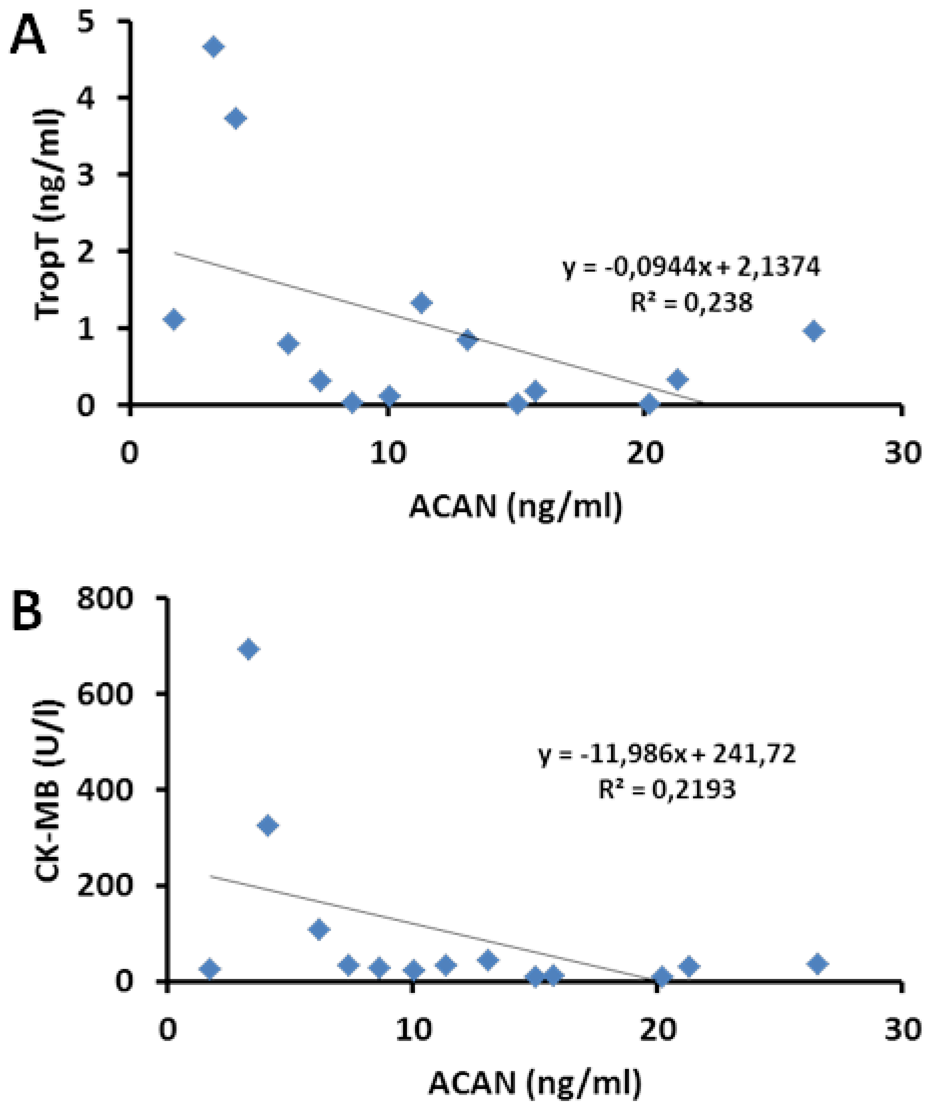

Supplementary Figure S3. ACAN serum protein level correlation with CK-MB or cTnT concentrations in patients with STEMI (N=18). A. Correlation between ACAN and CK-MB levels. **B.** Correlation between ACAN and cTnT levels. ACAN levels do not correlate with CK-MB or cTnT concentrations in patients with STEMI. ACAN, aggrecan, CK-MB, creatine kinase-muscle brain isoform, cTnT, cardiac troponin T.
